# Using a Clinical Decision Support System to Improve Anticoagulation in Patients with Nonvalve Atrial Fibrillation in China's Primary Care Settings: A Feasibility Study

**DOI:** 10.1155/2023/2136922

**Published:** 2023-01-18

**Authors:** Xueying Ru, Tianhao Wang, Lan Zhu, Yunhui Ma, Liqun Qian, Huan Sun, Zhigang Pan

**Affiliations:** ^1^Department of General Practice, Zhongshan Hospital, Fudan University, Shanghai 200032, China; ^2^Xuhui District Xietu Community Health Service Center, Shanghai 200023, China; ^3^Xuhui District Fenglin Community Health Service Center, Shanghai 200032, China; ^4^Pudong New Area Beicai Community Health Service Center, Shanghai 201204, China

## Abstract

**Background:**

To primarily investigate the effect of using a clinical decision support system (CDSS) in community health centers in Shanghai, China, on the proportion of patients prescribed guideline-directed antithrombotic therapy. This study also gauged the general practitioner (GP)'s acceptance of the CDSS who worked in the atrial fibrillation (AF) special consulting room of the CDSS group.

**Methods:**

This was a prospective cohort study that included a semistructured interview and a feasibility study for a cluster-randomized controlled trial. Eligible patients who sought medical care in the AF special consulting rooms in two community health centers in Shanghai, China, between April 1, 2020, and October 1, 2020, were enrolled, and their medical records from the enrollment date, up to October 1, 2021, were extracted. Based on whether the GPs in the AF special consulting rooms of the two sites used the CDSS or not, we classified the two sites as a software group and a control group. The CDSS could automatically assess the risks of stroke and bleeding and provide suggestions on treatment, follow-up, adjustment of anticoagulants or dosage, and other items. The primary outcome was the proportion of patients prescribed guideline-directed antithrombotic therapy. We also conducted a semistructured interview with the GP in the AF special consulting rooms of the software group regarding the acceptance of the CDSS and suggestions on the optimization of the CDSS and the study protocol of the cluster-randomized controlled trial in the future.

**Results:**

Eighty-four patients completed the follow-up. The mean age of these subjects was 75.71 years, the median time of clinical visits was six times per person, and the follow-up duration was 15 months. The basic demographics were similar between the two groups, except for age (*t* = 2.109, *p* = 0.038) and the HAS-BLED score (*χ*^2^ = 4.363, *p* = 0.037). The primary outcome in the software group was 8.071 times higher than that in the control group (adjusted odds ratio (OR) = 8.071, 95% confidence interval (2.570–25.344), *p* < 0.001). The frequency of consultation between groups was not significantly different (*p* = 0.981). It seemed that the incidence of adverse clinical events in the software group was lower than that in the control group. The main reason for dropouts in both groups was “following up in other hospitals.” The GP in the AF special consulting rooms of the software group accepted the CDSS well.

**Conclusions:**

The findings indicated that it was feasible to further promote the CDSS in the study among community health centers in China. The use of the CDSS might improve the proportion of patients prescribed guideline-directed antithrombotic therapy. The GP in the AF special consulting room of the software group showed a positive attitude toward the CDSS.

## 1. Background

Atrial fibrillation (AF) is one of the most common cardiac arrhythmia diseases [1]. There were more than 7.90 million patients with AF in China over 45 years of age, with a prevalence of 1.8%. Nearly 3% of people over 75 years of age had AF in China in 2020 [2, 3]. Due to the rapidly aging population, it was estimated that the prevalence of AF would increase at least 2.5 times in the next 50 years [4]. Thus, the increasingly high prevalence of AF brought a heavier disease burden on families and society. A systematic review published in 2011 indicated that the medical expense caused by AF management was 10,100–14,200 dollars per person in the United States and 450–3,000 euros per person in Europe [5]. Thromboembolic events, especially ischemic stroke, are the main issues of great expenses [1]. The risk of ischemic stroke in patients with AF was four to five times higher than that in patients without [3], with 24.2% of patients with nonvalve AF in China experiencing strokes [6].

As China progressed in primary care and implemented a two-way referral system, most patients with AF would receive antithrombotic medical services in community health centers in the future. The movement also corresponded with the view of “integrated care and stratified therapy” highlighted by some scholars, which meant that patients could get access to comprehensive management in primary care, such as risk assessment, AF treatment, international normalized ratio (INR) monitoring, health education, and treatment of comorbidity diseases, with superior hospitals responsible for the treatment of complications, emergencies, and operations [7]. However, at present, China's primary care settings are not equipped with sufficient capabilities and enthusiasm to undertake AF management [8, 9], due to heavy workloads already assigned to general practitioners (GPs) and the lack of knowledge of AF and anticoagulation treatment. Thus, many patients sought healthcare in superior hospitals or remained in unmanaged states [8]. For instance, the proportion of appropriate anticoagulation treatment in tertiary hospitals (the highest level of hospitals in mainland China) was 9.6%–68.4% but was only 4.0%–28.2% in nontertiary hospitals [6], and in the community health centers in Shanghai, the proportion dropped to 4.24%–12.64% [10–12]. To move AF management into primary care, the first step should be strengthening the GPs' AF management abilities. The development of clinical decision support systems (CDSSs) may help overcome such problems.

CDSSs, to some extent, are typical examples of artificial intelligence applications in healthcare settings, in which they are supported by computer algorithms. They can help primary care providers quickly make individualized and correct clinical decisions by combining patient data with guidelines in short consultations [13]. CDSSs significantly improved the GPs' work efficiency and quality, as well as reduced their workloads. As early as 2002 [14], scholars proposed the idea of applying CDSSs to AF management. AF antithrombotic management-associated CDSSs were then applied in some developed countries [15] and appraised by most GPs [16–18]. Due to the short follow-up duration, inadequate sample size, and imperfect design of CDSSs, many studies, however, reported that CDSSs might improve the appropriate prescription of antithrombotic agents and lower the incidence of adverse events but had no effects on reducing the incidence of thromboembolism [19–24]. Different from developed countries, the proportion of appropriate antithrombotic treatment in patients with AF is lower in China [25], and patients can seek medical care in any hospital they prefer. To our knowledge, no similar studies have been published in China's primary care settings until now.

We evaluated an AF antithrombotic management-associated CDSS, specifically designed for GPs, and wanted to carry out a cluster-randomized controlled trial to confirm its utility. This study was a prospective cohort study with a semistructured interview and a feasibility study for the cluster-randomized controlled trial. We aimed to primarily investigate the effect of the CDSS in community health centers in Shanghai, China, on the proportion of patients prescribed guideline-directed antithrombotic therapy. We also sought to evaluate the GP's acceptance of the CDSS who worked in the atrial fibrillation (AF) special consulting room of the CDSS group.

## 2. Methods

### 2.1. Study Design

This study was a prospective cohort study with a semistructured interview. We designed and reported the study according to the Strengthening the Reporting of Observational Studies in Epidemiology (STROBE) statement [26]. The Ethics Committee of Zhongshan Hospital in Shanghai, China, approved the study (approval number. B2022-009R).

### 2.2. Settings and Population

We selected the Fenglin Community Health Center as the software group, as it had eligible servers to install the CDSS and was willing to participate in the study. Considering the population of residents and the GP in AF special consulting rooms, we selected the Beicai Community Health Center, which is also in Shanghai, China, as the control group. The GPs in the two sites included were an associate chief physician and an experienced attending physician, respectively. Patients who sought medical care in the AF special consulting rooms of the two community health centers between April 1, 2020, and October 1, 2020, were potential participants. Patients who were older than 18 years of age, diagnosed with any type of AF or atrial flutter (ICD-10 codes are shown in Supplementary Material) during a consultation or within their medical history, and who were willing to participate in the study were included in this study. Individuals with valvular AF or prosthesis valves and those who were pregnant or breastfeeding were excluded.

### 2.3. Exposure

**Figure 1:**
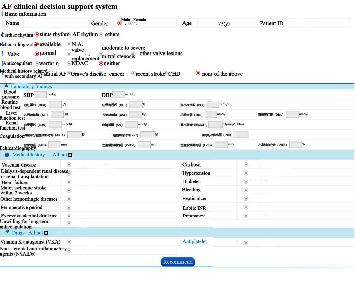
Interfaces of the CDSS for initial patients.

**Figure 2:**
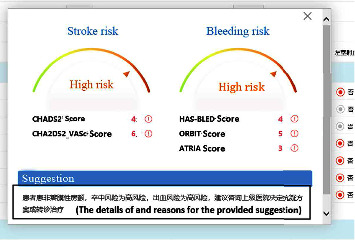
Examples of the treatment suggestions for initial patients.

**Figure 3:**
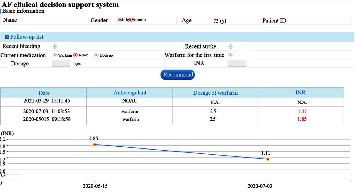
Interfaces of the CDSS for patient follow-up visits.

**Figure 4:**
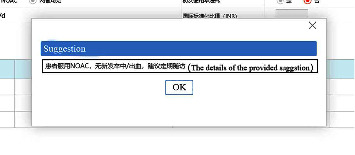
Examples of treatment suggestions during follow-up visits.

The exposure in this study was a CDSS for nonvalve AF anticoagulation management, which was developed by our team and Ping An Healthcare and Technology Co., Ltd., Beijing, China. The decision-making processes of the CDSS were developed based on the latest AF guidelines in China at that time [27]. Shenzhen Wang'an Computer Safety Checking & Measuring Technology Co., Ltd., Shenzhen, Guangdong Province, China, examined the system's security, and developers tested the stability and algorithm verification. The rationality of decisions was assessed by cardiological experts. Before use, we embedded the CDSS into the hospital information system (HIS) of the community health center in the software group. When one patient was diagnosed with AF during consultation for the first time, the GP opened the CDSS. After entering the patient's related information, such as personal details, medical or drug history, and laboratory or image findings (Figure 1), the CDSS evaluated the risk of strokes using the CHADS_2_ score and CHA_2_DS_2_-VASc score and the risk of bleeding using the HAS-BLED score, ORBIT score, and ATRIA score and provided treatment and follow-up suggestions according to the risks of the patient (Figure 2). Although the likelihood of program errors was low, we also added a red exclamation mark behind the scores of each risk evaluation (Figure 2). When the GP clicked the mouse on the red exclamation mark, they could see the explicit causes of these scores. During follow-up visits, the GP opened the follow-up visit interface of the CDSS and registered information, such as recent stroke and recent bleeding (Figure 3). The CDSS provided suggestions on whether to refer the patient to superior hospitals and how to adjust the dosage or frequency of anticoagulants (Figure 4). The GP then rendered final decisions according to those suggestions.

### 2.4. Outcomes

The primary outcome was the proportion of patients prescribed guideline-directed antithrombotic therapy. It was defined as the agreement percentage between the GPs' anticoagulation prescriptions for patients and the guideline's recommendations, including prescribing anticoagulation drugs to correct patients or recommending a referral to superior hospitals for patients with anticoagulation indications and refraining from anticoagulation for patients without such indications or with contraindications.

The secondary outcome was the frequency of consultation. It was defined as the average number of patients in each group, seeking medical care in community health centers due to AF. We also reported a composite of bleeding, stroke, death for AF or AF complications, and hospitalization for AF or AF complications. Strokes included ischemic stroke and transient ischemic attack (TIA). Referring to the Guidelines of Stroke Prevention in Chinese Patients with Atrial Fibrillation (2017), we classified bleeding events as minor bleeding and major bleeding [28]. When such adverse events occurred, we recorded the time of onset and ceased the follow-up.

### 2.5. Data Collection

We collected participants' medical records, from the enrollment date to October 1, 2021. We first extracted the demographic information of each subject from their health records, including their gender, age (y), systolic and diastolic pressure (mmHg), medical history, such as hypertension, diabetes, and cancer, current drugs, whether the patient was taking antiplatelet or nonsteroidal anti-inflammatory agents (NSAIDs), CHA_2_DS_2_-VASc score, HAS-BLED score, and anticoagulation contraindications if any.

How and when patients would revisit their GPs were determined by the subjects themselves after considering suggestions from their GPs. We extracted data on AF treatment from the subjects' medical records after the follow-up. The data collected included anticoagulants they used, INR values of patients with warfarin, and adverse clinical events during the follow-up. The occurrence of these events was identified according to their medical records, diagnostic records, and any related available auxiliary examinations. For some information that was not accessible via the Internet or due to loss to follow-up, data collection was performed by contacting the patients via telephone. At the end of the study, we interviewed the GP in the AF special consulting room of the software group to inquire about the acceptance of the CDSS and some rational improving ideas about the CDSS and the next cluster-randomized controlled trial. The interview lasted approximately 20 minutes. The interview was recorded (by audio and verbally) after obtaining informed consent from the GP. Two researchers summarized the conversations and independently extracted the key information related to the study. Disagreements were resolved through discussion or by a third researcher.

For subjects with new oral anticoagulants (NOACs), loss to follow-up was defined as the last follow-up visit date which was at least 12 months before the ending date (October 1, 2021). For patients receiving warfarin, that was defined as the last follow-up visit date was at least 1 month before the ending date [29].

### 2.6. Sample Size

According to the study by Whitehead et al. [30], the recommended sample size of a feasibility study was ten in each group, if the estimated effect in the formal research of a randomized controlled trial was ≥0.7 and the power (1-*β*) was 0.80.

### 2.7. Statistical Analysis

Categorical data were reported as frequencies and percentages (*n*, %), and chi-square tests were used to detect significant differences. Continuous data were reported as the mean ± standard deviation (SD) and compared using the *t*-test if they adhered to the Gaussian distribution. Otherwise, the data were reported as the median (interquartile range (IQR)) and compared using the Mann–Whitney *U* test. A logistic regression model was used to detect the effect of the exposure on the primary outcome. Considering the small sample size, we only calculated the number and incidence of composite adverse clinical events in each group and in total. To simplify the analysis, we recorded the follow-up time in the unit of month. Regardless of the start and end dates, we recorded the onset time as this month if events occurred in the former month or as the next month if they occurred in the latter. We presented the reasons for loss to follow-up in a pie diagram. All statistical analyses were performed using IBM SPSS Statistics, version 26.0 (SPSS Inc.). Two-tailed*p* < 0.05 was considered statistically significant.

## 3. Results

### 3.1. Basic Subject Demographics

**Figure 5:**
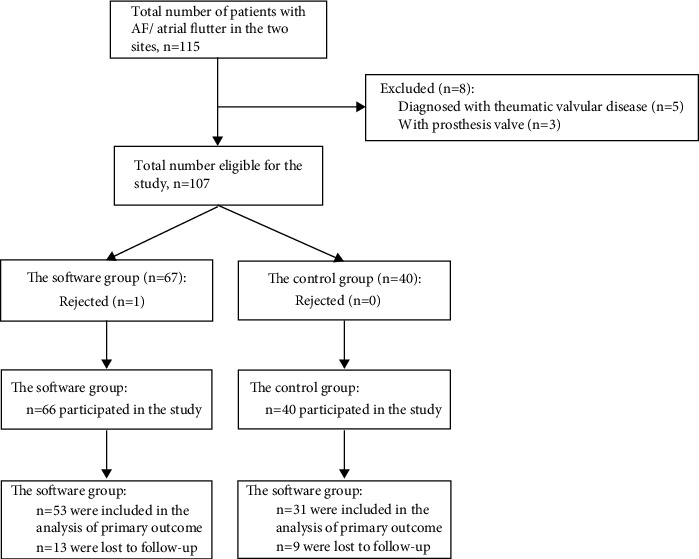
The study flowchart.

From April 1, 2020 to October 1, 2020, a total of 115 patients with AF or atrial flutter sought medical care in the AF special consulting rooms of the two community health centers. Eight patients met at least one of the exclusion criteria. Sixty-six subjects were in the software group, and 53 had complete records. Forty subjects were in the control group, and 31 had complete records. The flowchart of the study is shown in Figure 5.

Among the 84 subjects who completed the study, 46 (54.8%) were male. The average age was 75.71 years. The CHA_2_DS_2_-VASc scores of more than 95% of subjects were ≥2. The basic demographics were similar between the groups, except for age (79.96 ± 7.025 years in the software group and 73.58 ± 7.205 in the control group, *t* = 2.109, *p* = 0.038) and the HAS-BLED score (the number of subjects with the HAS-BLED score ≥3 was 33 in the software group and 12 in the control group, respectively; *χ*^2^ = 4.363, *p* = 0.037). The median time of clinical visits and the median follow-up duration were six times per person and 15 months, respectively, with no statistical differences between the two groups. The basic demographics of the subjects are shown in Table 1.

### 3.2. The Proportion of Patients Prescribed Guideline-Directed Antithrombotic Therapy

**Figure 6:**
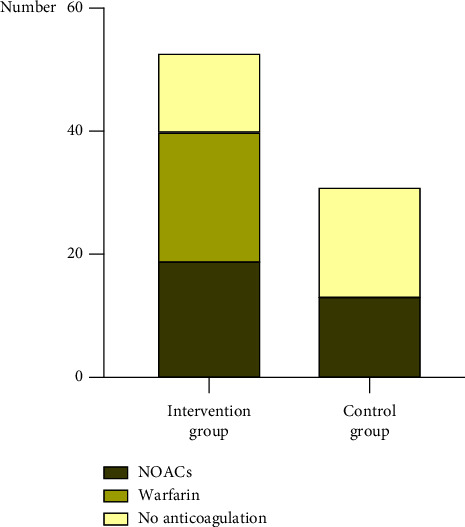
Histogram of antithrombotic treatment and the choice of anticoagulants in the two groups.

A total of 52 subjects in both groups received anticoagulants at the end of the study, with 40 in the software group and 12 in the control group. After removing subjects with anticoagulation contraindications, the proportion of patients prescribed guideline-directed anticoagulants was 75.5% in the software group and 38.7% in the control group. The only subject whose CHA_2_DS_2_-VASc score was lower than 2 but was prescribed with anticoagulants was in the software group, and his CHA_2_DS_2_-VASc score was one. In the software group, 19 individuals received NOACs and 21 received warfarin, while in the control group, all 12 individuals received NOACs (Figure 6).  Table 2 shows the factors associated with antithrombotic treatment. After adjusting for gender, age, hypertension, heart failure, diabetes, stroke/TIA/thromboembolism history, CHA_2_DS_2_-VASc score, and HAS-BLED score, the primary outcome in the software group was 8.071 times higher than that in the control group (adjusted OR = 8.071, 95% CI (2.570–25.344), *p* < 0.001), and the anticoagulation rate of patients receiving antiplatelets or NSAIDs was 0.165 times of those not receiving them (adjusted OR = 0.165, 95% CI (0.051–0.529), *p* = 0.002).

### 3.3. Adverse Clinical Events

Adverse events occurred in seven subjects during the study, and the incidence of all subjects was 8.3%. Among them, three were in the software group and four were in the control group. The incidence rates were 5.7% and 12.9% in the software group and the control group, respectively.

### 3.4. Causes of Participants Lost to Follow-Up

**Figure 7:**
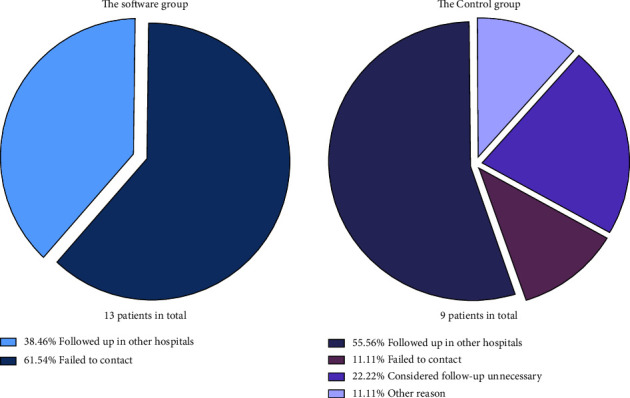
Pie diagram of the causes of participants lost to follow-up.

Twenty-two subjects were lost to follow-up, with 13 in the software group and nine in the control group. The dropout rate was observed with no statistical difference between the two groups. Significant differences were observed in the variables of stroke/TIA/thromboembolism history (*χ*^2^ = 4.935, *p* = 0.026), CHA_2_DS_2_-VASc score (*χ*^2^ = 6.034, *p* = 0.012), and HAS-BLED score (*χ*^2^ = 11.221, *p* = 0.001) (Table 3). Five subjects in each group reported that they followed up in other hospitals regularly, and two in the control group believed that they did not need to revisit for follow-up, as they experienced no uncomfortable symptoms (Figure 7).

### 3.5. The Qualitative Interview

Two topics were discussed during the qualitative interview, including the acceptance of the CDSS and suggestions to improve the CDSS and the protocol for the next cluster-randomized controlled study. The GP in the AF special consulting room of the software group accepted the interview.

### 3.6. Acceptance of the CDSS

The GP felt that the CDSS was helpful in her daily work and strengthened her confidence to prescribe necessary anticoagulants to patients. It was also useful when she taught patients about the precautions of anticoagulation. She also indicated that the CDSS might assist GPs with a poor command of AF management.

“*Connected with the hospital information system (HIS), the CDSS was convenient to use at any time. It helped me ranging from making a treatment decision for an initial patient to the treatment adjustment and referral in later follow-up, which made me more confident and effective in consultation with patients with AF. Although my hospital was near superior hospitals, many patients with AF still chose to seek medical care here. …… However, in recent consultations, I did not use the CDSS as frequently as before, as I generally became experienced in AF management and was sure of the decision made by myself.*”

### 3.7. Suggestions on the CDSS and the Study Protocol

This GP suggested adding assessments for the elderly as well as patients with comorbidity and modifying the methods for warfarin.

“*Despite the suggestions quickly provided by the CDSS, I sometimes still worry, especially for some elder patients or those with comorbidities. ……Since the formulation of warfarin in China is 2.5 mg per tablet, I need to convert the dosage for patients whose daily dosage was not 1 tablet.*”

## 4. Discussion

This study was a feasibility study of a cluster-randomized controlled trial which we are carrying out. The finding indicated that using the CDSS in China's primary care settings might improve the proportion of patients prescribed guideline-directed antithrombotic therapy. The GP in the AF special consulting room of the software group showed a positive attitude toward the CDSS.

Thromboembolic prophylaxis, through standardized anticoagulation, can significantly reduce the risk of strokes in patients with AF [31], as well as the elevated risk of disability and mortality caused by AF. Standardized anticoagulation plays an essential role in AF management. However, in China, the proportion of appropriate antithrombotic treatments administered to patients with AF was lower than that in most other countries [25]. This problem was even severe in primary care settings. One reason was that GPs in China lacked the knowledge to prescribe antithrombotic treatments and were worried about bleeding in patients with AF [9, 28].

The CDSS used in this study functioned to assess the risks of stroke and bleeding and provided suggestions on AF management, like treatment decisions, follow-up time, adjustment of anticoagulants, or dosage administered. The CDSS might help GPs manage patients with AF and enhance the GPs' capabilities and confidence in the field. Thus, it was well accepted by the GP in the AF special consulting room of the software group. The finding also indicated that the CDSS might improve the proportion of patients prescribed guideline-directed antithrombotic therapy and helped GPs provide scientific and normative health education to patients with AF. The proportion of appropriate anticoagulation treatments was 75.5% in the software group and 38.7% in the control group. There were 12 patients in the software group and 17 patients in the control group with high-stroke risks but were not prescribed with anticoagulants. The common reasons for no anticoagulation treatment in both groups were mainly old age, complex comorbidities, and strong rejection from patients themselves. Besides these, the GP in the control group also said that he was not sure if it was necessary to prescribe anticoagulants for those whose CHA2DS2-VASc scores were two and those who were taking antiplatelets or NSAIDs. Due to the few adverse clinical events during the study, we used a composite outcome indicator. It seemed that the incidence of adverse clinical events in the software group was lower than that in the control group, but this potential still needed to be further validated with a larger sample size in the cluster-randomized trial.

Considering the results in this study, we estimated that it should be feasible to promote the CDSS in more community health centers. In the subsequent randomized controlled study, we adjusted the protocol in the four aspects below. First, the dropout rate was high in the current study (21.5%), and one reason was that patients followed up in other hospitals. To reduce dropout rates, we will add inclusion and exclusion criteria to avoid the loss to follow-up. For example, we require that subjects be registered with a GP for at least 1 year and exclude patients whose expected survival time would be less than 1 year or those who are unable to cooperate with the study due to any reason. Second, considering that the GP in the AF special consulting room of the software group viewed the CDSS well, and patients seeking healthcare in AF special consulting rooms were few, we plan to carry out the formal trial in all general practice clinics. We want to recruit a sufficient number of participants and investigate whether using the CDSS remained effective or not in more community health centers. Therefore, in the next trial, we will set the inclusion and exclusion criteria for GPs and train them in AF management and the use of the CDSS. After training, GPs will be able to recruit patients with AF in the study. Third, based on the interview with the GP in the AF special consulting room of the software group, we will update the CDSS. Apart from its existing functions, the latest version also provides referral suggestions and tips on the interactions between anticoagulants and other drugs or food. The program also supports the assessments of the risk of falling and balancing (Tinetti Scale and Self-Rated Fall Risk Questionnaire), cognition (Mini-Mental State Examination), and emotional and psychological disorders (Geriatric Depression Scale-15 and the Confusion Assessment Method). In addition, we modified the record methods for the warfarin dosage. Fourth, GPs will be invited to complete a questionnaire on antithrombotic treatments three times before and after the training, as well as at the end of the study. Within the first week and the fourth, eighth, and twelfth months after enrollment, patients will be required to finish the satisfaction questionnaires. Through questionnaires, we hope to reduce dropouts to some extent and evaluate the effect of the CDSS in more directions. The revision for the protocol and the CDSS was approved by the Ethics Committee of Zhongshan Hospital and registered in the clinical trial registry (approval number. B2021-579(2) and registration number. ChiCTR2100052307) [32].

The study had some strengths. First, as far as we knew, the study was the first one in China to identify the effect of an AF antithrombotic treatment-related CDSS on patients with AF in primary care. Second, the data in this study were extracted from the medical records of patients or via telephone contacts. The data were reliable and close to the real environment of China's primary care. Third, the CDSS that we used was validated by algorithm engineers and cardiological experts and was highly praised by GPs in the AF special consulting room of the software group.

There also existed some shortcomings in this cohort study. First, the age and HAS-BLED score of the subjects at the baseline were not the same between the two groups. Second, the preferences for treatment choices of the subjects and GPs might influence the final treatment decisions, especially in regard to anticoagulant selection and for patients whose risk of stroke was one. However, we could not estimate the magnitude of such effects. Third, the data in this study were extracted from the medical records of each patient, and we did not have access to their socioeconomic information, such as their educational levels, incomes, and family support. However, in a previous study by Lunde et al. [33], the association between socioeconomic factors and AF decreased with increasing age. Fourth, the high dropout rate in this study might influence the results. However, in the two factors that were associated with the primary outcome in the study, we did not detect any difference between subjects who lost to follow-up and subjects who completed the follow-up. Fifth, the subjects were those who sought medical care in AF special consulting rooms of the two community health centers. The GPs in AF special consulting rooms should be more interested and experienced in AF anticoagulation treatment than most GPs. Therefore, the results in this study might not be able to be generalized to all GPs or all patients with AF in China's primary care.

## 5. Conclusions

From this study, we drew the primary conclusion that the use of the CDSS might improve the proportion of patients prescribed guideline-directed antithrombotic therapy. GPs in AF special consulting rooms of the software group had a positive attitude toward the CDSS. The finding indicated that it was feasible to further promote the CDSS in the study among community health centers in China, and a scientific, randomized, controlled trial was needed to verify the effectiveness of the CDSS in China's primary care.

## Figures and Tables

**Table 1:** Basic demographics of the subjects.

	Software group (*n* = 53)	Control group (*n* = 31)	Total (*n* = 84)	*χ* ^2^/*Z*/*t*	*p*
Gender, *n* (%)^$^				0.000	0.991
Male	29 (54.7)	17 (54.8)	46 (54.8)		
Female	24 (45.3)	14 (45.2)	38 (45.2)		
Age (y), mean (SD^&^)^#^	79.96 (7.025)	73.58 (7.205)	75.71 (7.237)	2.109	**0.038**
Hypertension, *n* (%)^$^	50 (94.3)	26 (83.9)	76 (90.5)	2.488	0.115
Heart failure, *n* (%)^$^	5 (9.4)	4 (14.8)	9 (11.3)	0.519	0.471
Diabetes, *n* (%)^$^	17 (32.1)	11 (35.5)	28 (33.3)	0.102	0.749
Stroke/TIA/thromboembolism history, *n* (%)^$^	13 (24.5)	3 (11.1)	16 (20.0)	2.013	0.156
Antiplatelets or NSAIDs, *n* (%)^$^	20 (37.7)	8 (29.6)	28 (35.0)	0.517	0.472
CHA_2_DS_2_-VASc score, *n* (%)^$^				1.183	0.277
≥2	52 (98.1)	29 (93.5)	81 (96.4)		
<2	1 (1.9)	2 (6.5)	3 (3.6)		
HAS-BLED score, *n* (%)^$^				4.363	**0.037**
≥3	33 (62.3)	12 (38.7)	45 (53.6)		
<3	20 (37.7)	19 (61.3)	39 (46.4)		
Clinical visits (times), median (IQR^*∗*^)^%^	7 (3, 16.5)	6 (4, 11)	6 (3, 13)	−0.023	0.981
Follow-up time (m), median (IQR^*∗*^)^%^	15 (13, 16.5)	16 (14, 17)	15 (13, 17)	1.209	0.227

*Note.* ^$^Chi-square test; ^&^standard deviation; ^#^two-tailed unpaired student's *t*-test;^*∗*^interquartile range; ^%^Mann–Whitney *U* test. Values in bold were statistically significant.

**Table 2:** Factors associated with the guideline-directed antithrombotic treatment of subjects.

	No.&/*n*	Crude OR (95% CI)	*p*	Adjusted OR^@^(95% CI)	*p*
Group (software/control)	40/12	4.872 (1.873–12.672)	**0.001**	8.071 (2.570–25.344)	**<0.001**
Gender (male/female)	32/20	2.057 (0.841–5.031)	0.114	—	—
Age	—	1.001 (0.941–1.064)	0.979	—	—
Hypertension	49	3.025 (0.671–13.644)	0.150	—	—
Heart failure	5	0.745 (0.184–3.006)	0.679	—	—
Diabetes	17	0.741 (0.293–1.87)	0.526	—	—
Stroke/TIA/thromboembolism history	11	1.449 (0.453–4.637)	0.532	—	—
Antiplatelets or NSAIDs	12	0.300 (0.116–0.773)	**0.013**	0.165 (0.051–0.529)	**0.002**
CHA_2_DS_2_-VASc score (≥2/<2)	51/1	3.400 (0.296–39.104)	0.326	—	—
HAS-BLED score (≥3/<3)	26/26	0.684 (0.281–1.667)	0.404	—	—

^&^Number of subjects with guideline-directed antithrombotic treatment; ^@^Adjusting for gender, age, hypertension, heart failure, diabetes, stroke/TIA/thromboembolism, CHA_2_DS_2_-VASc score, and HAS-BLED score. Values in bold were statistically significant.

**Table 3:** Basic demographics of subjects lost to follow-up.

	Completed follow-up(*n* = 84)	Loss to follow-up (*n* = 22)	Total (*n* = 106)	*χ* ^2^/*t*	*p*
Group, *n* (%)^$^				0.119	0.730
Software	53 (63.1)	13 (59.1)	66 (62.3)		
Control	31 (36.9)	9 (40.9)	40 (37.7)		
Gender, *n* (%)^$^				0.000	0.986
Male	46 (54.8)	12 (54.5)	58 (54.7)		
Female	38 (45.2)	10 (45.5)	48 (45.3)		
Age (y), mean (SD)^#^	75.71 (7.237)	75.91 (7.322)	75.75 (7.220)	−0.112	0.911
Hypertension, *n* (%)^$^	76 (90.5)	20 (90.9)	96 (90.6)	0.004	0.951
Heart failure, *n* (%)^$^	9 (11.3)	5 (22.7)	14 (13.2)	2.195	0.138
Diabetes, *n* (%)^$^	28 (33.3)	7 (31.8)	35 (34.0)	0.057	0.811
Stroke/TIA/thromboembolism history, *n* (%)^$^	16 (20.0)	0 (0.0)	16 (15.1)	4.935	**0.026**
Antiplatelets or NSAIDs, *n* (%)^$^	28 (35.0)	6 (30.0)	34 (32.7)	0.082	0.775
CHA_2_DS_2_-VASc score, *n* (%)^$^				6.034	**0.012**
≥2	81 (96.4)	18 (81.8)	99 (93.4)		
<2	3 (3.6)	4 (18.2)	7 (6.6)		
HAS-BLED score, *n* (%)^$^				11.221	**0.001**
≥3	45 (53.6)	3 (13.6)	48 (45.3)		
<3	39 (46.4)	19 (86.4)	58 (54.7)		

*Note.* ^$^Chi-square test; ^#^Two-tailed unpaired student's *t*-test. Values in bold were statistically significant.

## Data Availability

The datasets generated and/or analyzed in the current study are available from the corresponding author on reasonable request.

## Abbreviations

AF: Atrial fibrillation
CDSS: Clinical decision support system
CI: Confidence interval
GP: General practitioner
HIS: Hospital information system
HR: Hazard ratio
ICD-10: International Classification of Diseases
INR: International normalized ratio
IQR: Interquartile range
NOAC: New oral anticoagulant
NSAIDs: Nonsteroidal anti-inflammatory drugs
OR: Odds ratio
SD: Standard deviation
STROBE: Strengthening the Reporting of Observational Studies in Epidemiology
TIA: Transient ischemic attack.

## Supplementary Materials

Supplementary Materials ICD-10 codes of AF or atrial flutter.
