# Supplementary material for: Using a Clinical Decision Support System to Improve Anticoagulation in Patients with Nonvalve Atrial Fibrillation in China's Primary Care Settings: A Feasibility Study
Source: Int J Clin Pract. 2023 Jan 18;2023:2136922. doi: 10.1155/2023/2136922 (PMC9876694; doi:10.1155/2023/2136922)
Supplement: Supplementary Materials — ICD-10 codes of AF or atrial flutter. [file 2136922.f1.docx]

Supplemental file

**ICD-10 codes of AF and atrial flutter**

1. I48.200：chronic atrial fibrillation;
2. I48.100：persistent atrial fibrillation;
3. I48.900x015：first diagnosed atrial fibrillation;
4. I48.100x002：permanent atrial fibrillation;
5. I48.000：paroxysmal atrial flutter;
6. I48.100x003: long-standing persistent arial fibrillation;
7. I48.900x004：atrial fibrillation;
8. I48.900：atrial fibrillation or atrial flutter;
9. I48.900x003：atrial flutter;
10. I48.300：typical arial flutter;
11. I48.301：type I atrial flutter;
12. I48.400: untypical arial flutter;
13. I48.401：type II atrial flutter;
